# Racquet Mammoplasty as an Oncoplastic Technique in the Management of Lateral Quadrant Breast Cancer: A Prospective Controlled Study of Oncologic and Cosmetic Outcomes

**DOI:** 10.3390/medicina61030539

**Published:** 2025-03-19

**Authors:** Amr G. Mohamed, Emad M. Abdelrahman, Sherief M. Mohsen, Mostafa S. Abdeen, Mohamed A. Elsayed, Zizi M. Ibrahim, Osama R. Abdelraouf, Ebtesam N. D. Attia

**Affiliations:** 1 Department of General Surgery, Faculty of Medicine, Benha University, Benha 13518, Egypt; amr.gamal@fmed.bu.edu.eg (A.G.M.); ebtisam.attia@fmed.bu.edu.eg (E.N.D.A.); 2 Department of General Surgery, Faculty of Medicine, Ain Shams University, Cairo 11566, Egypt; dr.sheriefmohsen@outlook.com; 3 Plastic Surgery Unit, Department of General Surgery, Faculty of Medicine, Benha University, Banha 13518, Egypt; mustafa.abdeen@fmed.bu.edu.eg (M.S.A.); mohamed.elsayed@fmed.bu.edu.eg (M.A.E.); 4 Department of Rehabilitation Sciences, College of Health and Rehabilitation Sciences, Princess Nourah bint Abdulrahman University, P.O. Box 84428, Riyadh 11671, Saudi Arabia; zmibrahim@pnu.edu.sa; 5 Physical Therapy Program, Batterjee Medical College, Jeddah 21442, Saudi Arabia; pt4.jed@bmc.edu.sa

**Keywords:** breast cancer, oncoplastic breast surgery, racquet mammoplasty

## Abstract

*Background and Objectives*: The incorporation of oncoplastic surgery techniques into the management of breast cancer has become more popular and offers both oncological safety and good cosmetic results. However, it is challenging for surgeons to obtain good oncological control and acceptable cosmetic results. This study aims to evaluate racquet mammoplasty in patients managing lateral quadrant breast tumors. *Materials and Methods*: In total, 59 female patients with lesions in their lateral breast quadrants were operated upon using the racquet mammoplasty technique. Their intraoperative data and postoperative outcomes were assessed and analyzed. Follow-up was planned for at least 1 year later. *Results*: In the current study, 59 females were enrolled, with a mean age of 38.6 ± 4.3 years. Tumors were located in the lateral upper quadrant in most of the patients (74.4%). The mean size of the tumors was 2.7 ± 1.8. The surgical margins were free of malignancy in all cases. A total of 5.1% of cases reported a wound infection. A single patient developed loco-regional recurrence. Concerning the cosmetic outcomes, 89.8% of patients reported excellent outcomes and none reported poor or bad results. *Conclusions*: The racquet mammoplasty technique, when used as an oncoplastic technique, provides oncological safety as well as good cosmetic results for tumors in the lateral breast quadrants.

## 1. Introduction

Breast cancer is the most prevalent cancer in women in Egypt, accounting for 32% of all cancer cases according to data from the National Cancer Registry Program (NCRP), and its prevalence is predicted to triple by 2050 [1]. Mastectomy has been linked to the lowest quality of life evaluations because it results in physical and physiological mutilation, which exacerbate depressive symptoms [2]. Sentinel lymph node biopsy (SLNB) in place of traditional axillary clearing and breast-conserving therapy in place of mastectomy are two novel procedural advancements in breast cancer surgery that are thought to improve the patient’s quality of life without compromising their prognosis [3]. Meticulous evaluation of the oncological requirements and the esthetic goals is necessary as these are the primary paradigms used in oncoplastic breast surgery [4].

In addition to being a crucial component of surgical treatments for breast cancer, oncoplastic breast surgery (OBS) has an extended role in breast-conserving therapy (BCT), which is provided to patients who do not need a mastectomy to achieve satisfactory tumor clearance [5].

In order to improve cosmetic outcomes and make use of innovative procedures without compromising oncological safety, surgeons are constantly upgrading and modifying OBS approaches [6]. Oncoplastic surgery can be categorized into different levels according to the volume of tissue required to be excised. Either volume displacement or replacement techniques can be utilized to fill the breast cavity and compensate for the volume of tissue excised [7]. The tumor’s location and the volume of the excision are the main factors affecting the selection of the procedure. Breast, shape, and patient preferences are other breast characteristics that need to be taken into account [8]. Nevertheless, in certain instances, the tumor cannot be removed without causing cosmetic deformities and compromising the esthetic outcome [9].

The location of the tumor influences the oncoplastic surgical technique chosen. Less than 20% of the breast volume is removed during BCS in tiny-to-moderately sized breasts with little ptosis, which is known as level I oncoplastic breast surgery. On the other hand, level II oncoplastic breast surgery is characterized by the partial mastectomy of moderately sized-to-big breasts with moderate-to-severe ptosis which necessitates the excision of 20% to 50% of the breast tissue. Skin excision for reshaping and mammoplasty is not required if the maximum excision volume of the breasts is less than 20%. Many oncoplastic surgery techniques have been used for outer quadrant breast cancer lesions, including lateral mammoplasty, Round block, inferior pedicle reduction, Superior pedicle reduction, J mammoplasty, and single-incision lateral sulcus mammoplasty [10].

Improving the cosmetic shape post-operation is a crucial aspect of rehabilitation for breast cancer survivors. Physical therapy plays a vital role in restoring function, symmetry, and esthetic appearance. Key techniques here include scar tissue management, postural correction, strengthening exercises, and upper-body core stabilization, all of which contribute to improved mobility and overall cosmetic outcomes [11,12].

In this work, we describe the use of racquet mammoplasty to treat breast cancers in the lateral breast quadrant, a location where breast cancer frequently occurs and where breast-conserving surgery has been frequently associated with poor esthetic effects, Nipple–areola complex (NAC) deviation, and deformity exaggerated by postoperative radiotherapy.

The aim of this study was to assess the use of racquet mammoplasty as an oncoplastic technique in the management of lateral quadrant breast cancer, with the expectation of good cosmetic results.

## 2. Materials and Methods

### 2.1. Study Design

The current study was conducted following the ethical guidelines of Helsinki Declaration. This study’s design was approved by an ethical committee and informed written consent was obtained from all participants for their inclusion in the study after discussion and education about the technique. The present multicenter study was conducted in the General Surgery departments in Benha and Ain Shams Universities throughout the period from January 2021 to December 2024. This study included 59 female patients who were diagnosed with breast cancer in the lateral quadrants of their breast and were motivated and eligible for oncoplastic breast surgery in the form of racquet mammoplasty.

All the patients were subjected to history-taking, a clinical breast and axillary examination, and bilateral sono-mammography. The malignant nature of the afflicted lesion was established by a true-cut needle biopsy.

The inclusion criteria were patients with unilateral, unifocal pathologically proven breast cancer in the lateral breast quadrants with a relatively large tumor in relation to their breast volume. The stages of the tumors included were T1, T2, and T3 after down-staging with neoadjuvant chemotherapy and preoperative wire localization.

Exclusion criteria were patients with T4 tumors or patients with distant metastasis. Lesions less than 1 cm proximal to the nipple–areolar complex, multicentric tumors, inflammatory breast tumors, and patients with diffuse microcalcifications were also excluded from the study. 

### 2.2. The Surgical Procedure

#### Markings

**Figure 1:**
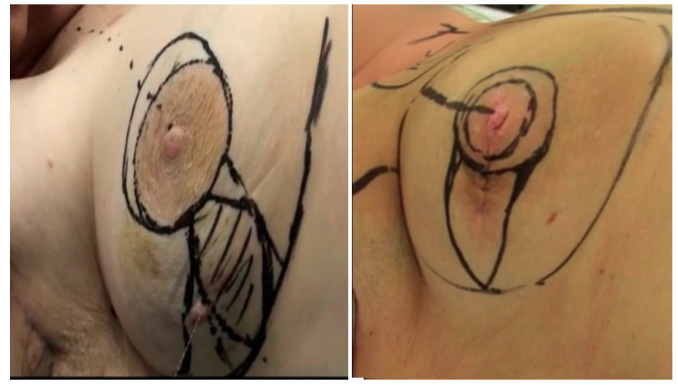
Preoperative markings showing the ellipse encircling the tumor and the lines to the areola and peri-areolar area according to the site of the lesion.

While the patient is standing, preoperative markings are created. First, an encircling safety margin is used to indicate the location of the tumor in the breast (Figure 1). Both the surrounding peri-areolar de-epithelization region and the NAC are marked. An ellipse around the previously marked tumor is drawn from the NAC to the inframammary fold (IMF) with equal limbs.

**Figure 2:**
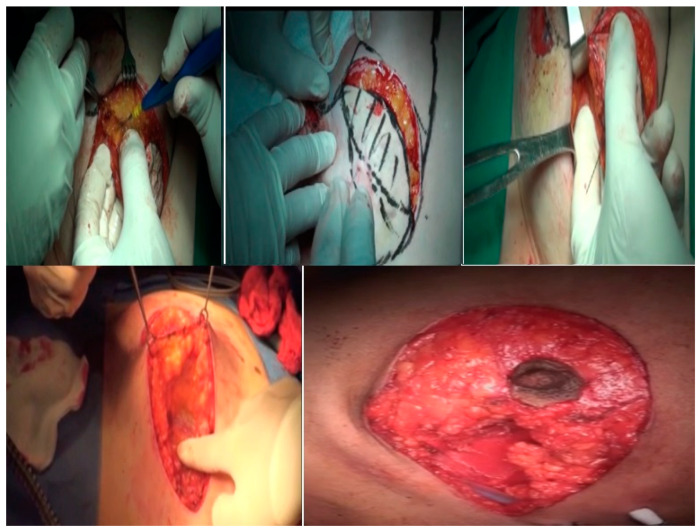
Dissection and excision of the tumor, with bimanual palpation down to the pectoral fascia.

Skin incision and breast resection: Patent blue dye is injected in the sub-areolar region if a SLNB is planned. Peri-areolar de-epithelization of the peri-areolar area is performed in a circumferential manner. The excision of the ellipse of skin encircling the tumor is performed down to the pectoral fascia using the bimanual palpation of the tumor, and then the specimen is sent for a histopathological evaluation of its margins (Figure 2). A re-excision is performed if there is a positive surgical margin. The tumor bed is marked with clips to facilitate postoperative radiotherapy.

**Figure 3:**
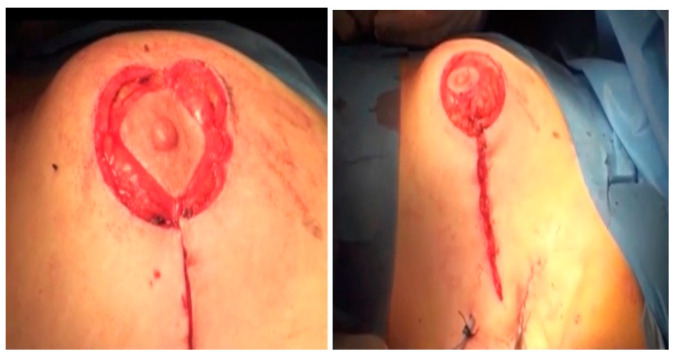
NAC repositioning and closure of the incision.

**Figure 4:**
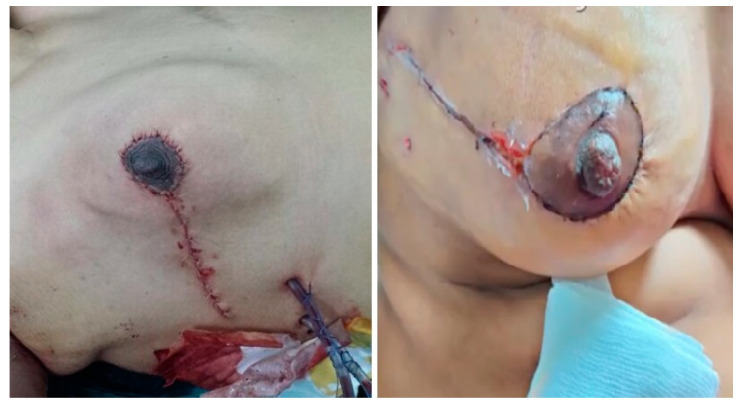
Postoperative view after one week.

Axillary surgery is performed in the form of an SLNB and, in cases of a positive SLNB axillary dissection, a level I or II axillary lymph node dissection (ALND) is performed. Axillary surgery is performed using the same incision or a separate axillary incision whenever needed. The NAC is repositioned to its new position on a reliable central pedicle to maintain vascularity and sensation (Figure 3 and Figure 4). The clavipectoral fascia is closed again to separate the breast cavity from the axillary cavity, if an axillary lymphadenectomy was performed from the same breast incision, keeping the glandular seroma desired for breast reshaping. Central, lateral, and medial dermo-glandular flaps are approximated to close the defect. A drain is left in the cavity if needed after proper glandular approximation and another limb of the drain is left in the axilla if an axillary dissection is performed. Contralateral breast summarization was carried out for some patients who sought mastopexy and summarization. This was performed through the peri-areolar de-epithelization of the peri-areolar area in a circumferential manner as a minor mastopexy near the pathological side.

**Figure 5:**
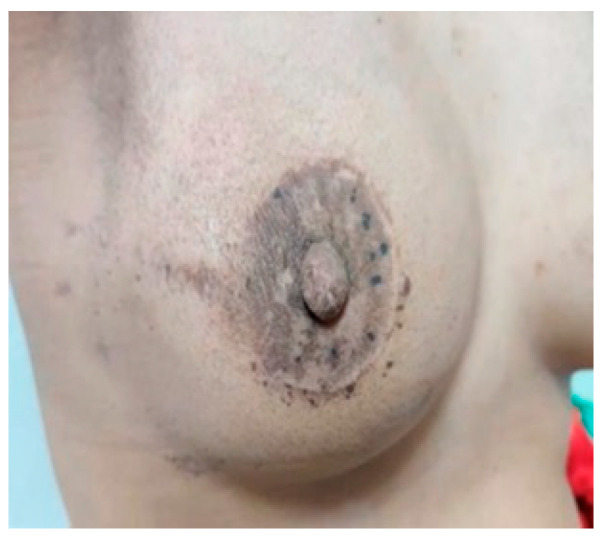
Final cosmetic outcomes after 3 months.

Post-operation: The patients are reviewed in the outpatient clinic 1 week and 2 weeks after the surgery for the assessment of wound complications and the final esthetic outcome (Figure 5). Follow-ups are scheduled every 4 months for the first two years, then every 6 months for 3 years, and then annually.

All the patients received postoperative radiotherapy and then additional adjuvant therapy such as chemotherapy or hormonal or target therapy according to our standard institutional protocols, with a physiotherapy intervention provided to manage treatment-related sequelae such as post-mastectomy pain, upper limb dysfunction, and cancer-related fatigue [13].

### 2.3. Follow-Up and Outcomes

The primary outcome was the excision of outer quadrant breast masses using a racquet mammoplasty for oncological safety with limited postoperative complications.

The secondary outcome was achieving accepted esthetic outcomes and patient satisfaction.

All the patients were assessed with regard to the duration of the procedure, the status of their resection margins, and morbidity (wound infection or dehiscence, hematoma, seroma, NAC and skin slough or necrosis, and NAC sensation).

The cosmetic outcome was assessed by asking the patient herself to rate the result of the surgery in terms of breast symmetry, scarring, and her degree of satisfaction using the Likert scale [14], which was simply presented as a five-point score (1 = excellent, 2 = good, 3 = fair, 4 = poor, and 5 = bad). On the other hand, the esthetic outcome was obtained by the assessment of the breast’s symmetry, position, deformity in the NAC, scarring, retraction, and the final scar’s appearance. This was carried out by three independent plastic surgeons using Vancouver’s scar scale (VSS) [15,16] (Table 1), The total score ranges between 0 (normal skin) and 13 (the worst imaginable scar).

### 2.4. Statistical Analysis

G*power 3.1 program (Dusseldorf, Germany’s) was used to estimate the sample size required. Taking into consideration a 20% dropout rate during follow-up, 59 patients were recruited, with an effect size of 0.9, 95% power, and a 0.05 type-one error.

Student’s *t*-test was used to conduct statistical analyses for quantitative parameters that were described by a mean and SD. For qualitative data that were expressed as frequencies with a percent, the χ^2^ test was employed. (IBM SPSS) software, version 21.0 (2013; IBM Corp., Chicago, IL, USA), was employed. A significance threshold of less than 0.05 was applied to probability values.

## 3. Results

In the current study, 59 female patients with invasive breast cancer in their lateral breast quadrants were subjected to the racquet mammoplasty technique. The mean age of the study group was 38.6 ± 4.3 years and their mean body mass index (BMI) was 29.6 ± 3.2 kg/m^2^. The majority of the patients were medically free and only 10.2% of the eligible patients were diabetic. Other sociodemographic data are shown in Table 1.

Table 1 reported that the tumors were located in the lateral upper quadrant in most of the patients (74.4%). The mean size of the tumors was 2.7 ± 1.8. The mean distance between the tumor and the NAC was 3.52 ± 0.83. According to the TNM classification system, T1 tumors were found in 55.9% of cases. The majority of the patients had invasive ductal carcinoma (94.9%).

The mean operative time of the procedure was 112.6 ± 23.2 min. The surgical margins were free in all cases and no cases required re-excision. The mean weight of the excised specimen was 61.3 ± 12.4 gm. All the patients underwent SLNB which proved to be negative in 30.5% of cases, while positivity was reported in 69.5%, following which level I and II axillary dissections were completed (Table 2).

The mean duration of the follow-up was 14 months and ranged between 12 and 18 months. No significant complications were encountered in the postoperative period apart from 5.1% of cases reporting a wound infection, which was managed conservatively. No marginal skin or NAC necrosis occurred in any of our patients and NAC sensation was preserved in all cases. A single patient developed loco-regional recurrence in their ipsilateral breast, in the upper outer quadrant, at 12 months of follow-up and was managed with a salvage mastectomy. None of the patients developed distant metastasis during the follow-up period of this study (Table 2). Although 6.8% of patients developed asymmetry, none of them asked for symmetrization.

Concerning the cosmetic outcomes, the results, as assessed by the patients, were excellent in 89.8% of patients, good in 4 (6.8%) patients, fair in 2 (3.4%) patients, with no poor or bad results reported. The reported mean VSS was 2.3 ± 0.89 (Table 3).

## 4. Discussion

With a survival rate comparable to mastectomy, BCT, which involves the broad local excision of tumors followed by radiation therapy, is rapidly becoming a recognized treatment for breast cancer. Furthermore, it improves quality of life and body contouring [17,18]. The advent of OBS has been crucial to the advancements made in breast cancer surgery, improving patient quality of life [19].

Surgeons were able to expand the use of BCT while preserving oncological safety and esthetic results because of OBS. Larger resection margins and improved cosmetic outcomes were made possible by the extension of quadrantectomies with the use of plastic surgery techniques [20].

There are three main targets of OBS: safe oncological resection, immediate simultaneous reconstruction using volume displacement or replacement techniques, and contralateral breast symmetry [21].

Perhaps one of the hallmark achievements of OBS is good resection margins, avoiding the risk of local recurrence. Many previous studies suggest that there is a low incidence of positive margins in OBS compared with conventional BCT [22].

Here, we introduce the technique of racquet mammoplasty as a useful oncoplastic technique for lateral quadrant breast tumors. The most commonly used technique for such tumors has been the inverted T-Wise pattern technique and either the inferior or superior pedicle technique, according to position of the tumor, which allows a wide resection both in the vertical and horizontal directions. However, this technique is associated with a lot of scarring and is more technically demanding [22].

In our technique, we utilized only a laterally placed incision, which is simple to implement and does not require a long learning curve.

It is worth mentioning that all our patients achieved negative resection margins upon frozen section assessment and none of them needed re-excision.

We believe that the importance of racquet mammoplasty in tumors following their down-staging via neoadjuvant therapy is increasing. In our study, 29 (49.1%) patients received neoadjuvant chemotherapy and preoperative wire localization and were fit for the technique, with good esthetic scores achieved afterward. Much evidence exists demonstrating the safety of BCT following a clinical response after neoadjuvant therapy [22].

In our study, the rate of postoperative complications was very low. Only 5.1% of patients developed a wound infection, and these were managed conservatively and did not delay the adjuvant therapy. This result is better than that in other studies on oncoplastic breast surgery, for which the complication rate may reach up to 20% [23], and this can be simply explained by the relatively easier technique used in the current study. It should be noted that different oncoplastic techniques differ in the extent of their resection and the extent of their dissection, making comparisons problematic.

In a series of 540 patients who underwent different oncoplastic techniques, extensive tissue undermining increased the rate of seroma, fat necrosis, and total or partial NAC necrosis [24]. In our study, only one patient developed such complications.

Our technique was ideal in patients with moderately to large-sized breasts with mild ptosis. The extent of the resection of normal breast parenchyma in our technique was less than other oncoplastic reduction techniques that focus mainly on the tumor itself and we were able to obtain free resection margins. The mean weight of the excised specimens was 61.3 ± 12.4 gm. None of the patients in our study required contralateral summarization even after the completion of the postoperative radiotherapy course, although four patients reported mild asymmetry. However, they accepted the outcome and did not opt for delayed contralateral breast surgery, which is in contrast to many authors [25,26] who recommend delayed contralateral reduction after radiotherapy due to the anticipation of a reduction in the breast volume and more asymmetry.

No patients developed distant metastasis during the follow-up of this study and only one patient (1.7%) developed local recurrence in their ipsilateral breast, in the upper outer quadrant; these results are better than those of other authors [23], who reported a local recurrence rate of 6.8%, and this may be due to the relatively short period of follow-up in the present study.

These results emphasize the safety of the technique, which enables the obtainment of good and adequate resection margins. This may be attributed to the utilization of the bimanual palpation technique during resection and the importance of intraoperative frozen section examination. Careful patient selection may be a factor, as we did not include patients with diffuse microcalcification or multifocal lesions. No delay in adjuvant therapy or wound healing was reported.

Concerning the cosmetic outcomes, excellent results were observed in 89.8% of patients. No poor or bad results were reported. No revisional surgery was required on an esthetic basis.

Further studies may be required to assess the feasibility and safety of this technique in cases of diffuse microcalcification and multifocal lesions. Moreover, a comparison of the results with other oncoplastic techniques may be beneficial.

### Limitation

The relatively smaller number of previous well-designed studies to compare our results with was the main limitation of this study.

## 5. Conclusions

Racquet mammoplasty is a novel oncoplastic technique used in tumors in the lateral quadrants of the breast, especially in females with moderately sized to large breasts. It has the advantages of being simple, reliable, oncologically safe, and cosmetically acceptable.

## Figures and Tables

**Table 1:** Patients’ demographic data and tumor characteristics.

Variable		N = 59
Age	Mean ± SD	38.6 ± 4.3
BMI (kg/m^2^)	Mean ± SD	29.6 ± 3.2
Comorbidities		
Diabetes mellitus	N (%)	6 (10.2%)
Hypertension	N (%)	9 (15.2%)
Ischemic heart disease	N (%)	3 (5.1%)
Tumor characteristics		
Tumor size	Mean± SD	2.7 ± 1.8
Tumor location		
Lateral upper quadrant (LUQ)	N (%)	44 (74.6%)
Lateral central quadrant (LCQ)	N (%)	9 (15.2%)
Lateral lower quadrant (LLQ)	N (%)	6 (10.2%)
Distance between tumor and NAC (cm)	Mean± SD	3.52 ± 0.83
Pathological tumor type		
Invasive ductal carcinoma	N (%)	56 (94.9%)
Invasive lobular carcinoma	N (%)	3 (5.1%)
TNM classification		
T1	N (%)	33 (55.9%)
T2	N (%)	24 (40.7%)
T3	N (%)	2 (3.4%)
N0	N (%)	25 (42.4%)
N1	N (%)	34 (57.6)
N2	N (%)	0 (0%)
M0	N (%)	59 (100%)
M1	N (%)	0 (0%)
Neoadjuvant chemotherapy (n)(%)	N (%)	29 (49.1%)

**Table 2:** Operative findings and postoperative sequelae.

Variable		N = 59
Mean operative time (min)	Mean ± SD	112.6 ± 23.2
Weight of excised specimen (gm)	Mean ± SD	61.3 ± 12.4
Axillary surgery		
Sentinel lymph node biopsy	N (%)	18 (30.5%)
Axillary dissection (level I and II)	N (%)	41 (69.5%)
Postoperative complications		
Early complications		
Wound infection	N (%)	3 (5.1%)
Hematoma	N (%)	1 (1.7%)
Seroma	N (%)	1 (1.7%)
Marginal skin necrosis	N (%)	0 (0%)
NAC necrosis Late complications	N (%)	0 (0%)
Impaired NAC sensation	N (%)	0 (0%)
Asymmetry	N (%)	4 (6.8%)
Fat necrosis	N (%)	2 (3.4)
Local recurrence	N (%)	1 (1.7%)
Distant metastasis	N (%)	0 (0%)
Adjuvant therapy		
Radiotherapy	N (%)	59 (100%)
Chemotherapy	N (%)	48 (81.4%)
Hormonal therapy	N (%)	51 (86.5%)

**Table 3:** Assessment of cosmetic outcomes.

Variable		N = 59
Likert Scale		
Excellent	N (%)	53 (89.8%)
Good	N (%)	4 (6.8%)
Fair	N (%)	2 (3.4%)
poor	N (%)	0 (0%)
Bad	N (%)	0 (0%)
Vancouver Scar Scale	Range	1–6
	Mean ± SD	2.3 ± 0.89

## Data Availability

The data from this work are available from the authors and not published before elsewhere.
